# Characteristics of Studies Focusing on Vaccine Series Completion Among Children Aged 12–23 Months in Sub-Saharan Africa: A Scoping Review

**DOI:** 10.3390/children12040415

**Published:** 2025-03-26

**Authors:** Weiqi Li, Fabrice Sewolo, Andrew Aoun, Minyahil Tadesse Boltena, Amro Musad, Ann Lindstrand, Tobias Alfvén, Claudia Hanson, Ziad El-Khatib

**Affiliations:** 1 Department of Global Public Health, Karolinska Institutet, 17177 Stockholm, Sweden; weiqi.li@charite.de (W.L.); tobias.alfven@ki.se (T.A.); claudia.hanson@ki.se (C.H.); 2 Field Epidemiology Training Program (FETP), Kinshasa 1004131, Democratic Republic of the Congo; fsewolo@gmail.com; 3 Faculty of Health Sciences, University of Ottawa, Ottawa, ON K1H 8M5, Canada; aaoun013@uottawa.ca; 4 Ethiopian Evidence Based Health Care Centre: A Joanna Briggs Institute Center of Excellence, Faculty of Public Health, Institute of Health, Jimma University, Jimma P.O. Box 378, Ethiopia; minyahil.tadesse@ahri.gov.et; 5 Knowledge Translation Division, Knowledge Management Directorate, Armauer Hansen Research Institute, Ministry of Health Addis, Ababa P.O. Box 1234, Ethiopia; 6 Department of Learning, Informatics, Management and Ethics (LIME), 17177 Stockholm, Sweden; amro.musad@hotmail.com; 7 Department of Immunization, Vaccines and Biologicals, World Health Organization, 1211 Geneva, Switzerland; lindstranda@who.int

**Keywords:** childhood vaccination, series completion, Sub-Saharan Africa

## Abstract

Vaccine preventable diseases remain the leading causes of death among children in Sub-Saharan Africa. Background/Objectives: As vaccines provide the best possible protection only when all required doses are received, it is essential to understand how the published literature is designed and conducted regarding the completion of recommended childhood vaccine series for children aged 12–23 months in SSA. Methods: A comprehensive search was conducted across five databases (PubMed, Embase, CINAHL, Web of Science, and Google Scholar) to identify the relevant literature published between January 2000 through December 2023. Results: A total of 53 studies meeting the inclusion criteria were identified from the five databases. Over half of the studies used a cross-sectional design (*n* = 32, 60.4%), and less than half of the studies were conducted in Ethiopia (*n* = 23, 43.4%). The prevalence and associated factors of vaccination series completion were the most commonly explored topics in the literature. The most frequently reported factors associated with vaccine series completion included the caregiver’s education level, household wealth status, number of children under five in the household, knowledge of immunization, maternal education, place of residence, gender of the household head or decision maker, utilization of antenatal or postnatal care visits, place of delivery, distance to a healthy facility or travel time, and possession of a vaccination card. Conclusions: This scoping review identified methodological gaps in the published literature, including a lack of publications from many Sub-Saharan Africa countries and insufficient evidence on trends and inequalities in vaccine series completion. Future research on vaccine series completion is recommended to address these gaps.

## 1. Introduction

Vaccine series completion (VSC) is essential for preventing vaccine-preventable diseases (VPDs) and reducing child mortality worldwide. Over the past five decades, global vaccination coverage has increased significantly, driven by initiatives such as the Immunization Agenda 2030 (IA2030). The Expanded Program on Immunization (EPI), recognized for its cost-effectiveness, has substantially decreased childhood morbidity and mortality on a global scale [1]. For instance, the coverage of the third dose of diphtheria–tetanus–pertussis-containing vaccine (DTP3) rose from 79% in 2007 to 84% in 2022, and over 80% of children received the first dose of the measles-containing vaccine (MCV1) by their second birthday in 2022 [2]. Despite these achievements, VPDs still account for approximately 17% of global annual mortalities in children under five years of age [3]. The resurgence of measles in 2024 underscores the persistent gaps in immunization coverage [4]. The coronavirus disease (COVID-19) pandemic further impacted global vaccine coverage, resulting in a 3% decline from 2019 to 2020 and an increase in the number of unvaccinated children (zero-dose) from 12.9 million in 2019 to 14.3 million in 2022 [2]. However, VSC, defined as the completion of all vaccination doses at multiple visits within a specified period of time, has been proven to be a more robust indicator for assessing childhood immunization progress. It has been reported as “all age-appropriate vaccinations” in many Demographic Health Surveys (DHSs) conducted in Sub-Saharan Africa (SSA) countries [5]. Since the implementation of the Expanded Program on Immunization (EPI) in SSA in 1978, efforts to enhance vaccination coverage have been hindered by persistent systemic challenges [6]. These challenges include limited resources, unequal access to vaccines, and inadequate healthcare infrastructure. In 2016, approximately 20% of children in SSA missed essential vaccinations, resulting in over half a million annual deaths attributable to VPDs. Furthermore, by the end of the last decade, 60% of countries, most of them in SSA, did not achieve the target of 90% coverage for all scheduled vaccines [6].

Given the high risk of VPD outbreaks, generating robust and reliable estimates of vaccination coverage is critical for guiding and optimizing immunization strategies in SSA [7]. Current research on childhood VSC in SSA reveals significant gaps and inconsistencies. Notable barriers include inadequate parental knowledge, vaccine hesitancy, and systemic obstacles such as disrupted cold chains and insufficient healthcare provider training [8,9,10]. However, the extent to which studies focus on the proportion of children receiving all compulsory doses recommended by national immunization programs (NIPs) remains unclear. Furthermore, no prior research has systematically aggregated and evaluated these studies at a regional level within SSA. This lack of comprehensive evaluations underscores the urgent need for detailed studies to inform immunization strategies and address gaps that hinder progress toward the World Health Organization (WHO) Immunization Agenda 2030 (IA2030) and Sustainable Development Goals (SDGs).

This scoping review aimed to identify and synthesize the existing literature on VSC among children aged 12–23 months in SSA. By characterizing the available research, this review seeks to identify research gaps and advance understanding in the field.

## 2. Materials and Methods

### 2.1. Search Strategies

The study was conducted following the Preferred Reporting Items for Systematic Reviews and Meta-Analyses (PRISMA) guidelines (Supplementary Section S1) [11]. A search was conducted in five scientific databases: PubMed, Embase, CINAHL, Web of Science, and Google Scholar. The search period ranged from 29 January 2024 through 30 March 2024. The following combination of keywords was used: (“immunization OR vaccination”) AND (“coverage” OR “uptake” OR “completion” OR “rate” OR “ proportion”) AND (“children” OR “childhood” OR “12–23 months” OR “under 2 years” OR “toddlers”) AND (“Sub-Saharan Africa” OR “Angola” OR “Benin” OR “the name of the rest 46 countries” OR (“East Africa”) OR (“Middle Africa”) OR (“Southern Africa”) OR (“West Africa”) OR (“Central Africa”) OR (“Western Sahara”). The search strings were tailored to meet the specific requirement of each database (Supplementary Section S2).

### 2.2. Eligibility Criteria

To ensure the methodological quality and alignment with the WHO-recommended vaccine list for global immunization programs, inclusion and exclusion criteria were applied during the scoping review.

Inclusion criteria:

- Primary research studies (quantitative, qualitative, or mixed-methods) and the gray literature conducted in SSA, whether covering part of a country, an entire country, or multiple countries.
- Focused on children aged 12–23 months.
- Reported the VSC rates, as defined in this study.
- Published in English between January 2000 and December 2023, coinciding with the introduction of pentavalent, pneumococcal conjugate (PCV), and rotavirus (RV) vaccines in most SSA countries.

Exclusion criteria:

- Lacked a clear definition of VSC or relevant concepts.
- Did not provide specific VSC rates or related metrics.
- Focused exclusively on one or several types of vaccines rather than the full immunization schedule or included vaccines not part of the national EPI.
- Were systematic reviews, study protocols, journal commentaries, or conference papers.
- Were duplicate studies or lacked access to full texts.

All articles identified during the search were imported into Endnote 20 for screening. After de-duplication, the titles and abstracts of retrieved articles were reviewed to identify potentially relevant studies, followed by full-text screening to determine final inclusion for further analysis. A random sample of 10% of the articles was screened by two reviewers to ensure consistency (WL and AA). The remaining articles were screened by one reviewer (WL), with conflicts addressed by a second reviewer (AA) and uncertainties resolved by a third reviewer (ZEK).

### 2.3. Data Extraction and Analysis

**Figure 1:**
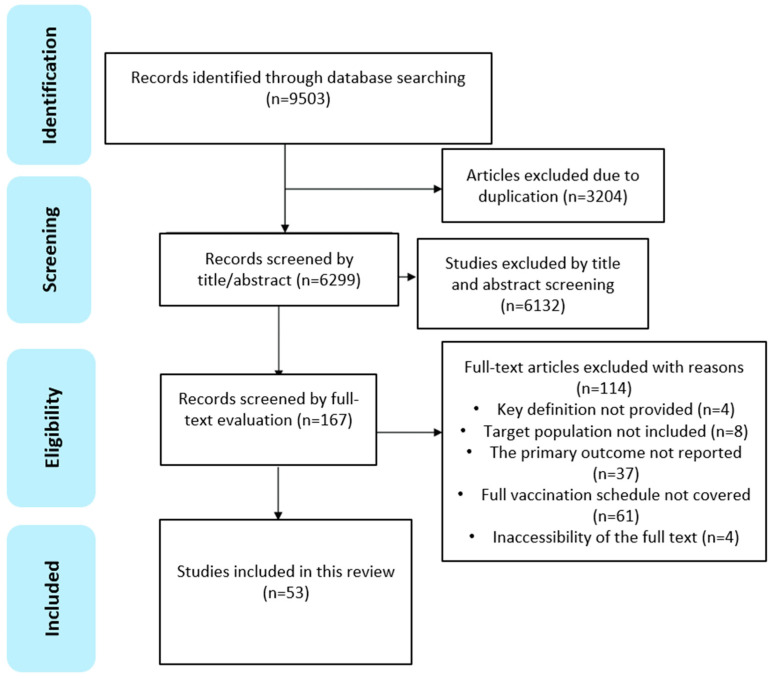
PRISMA flowchart indicating the process of study identification, screening, and inclusion.

The study’s data extraction and analysis followed a two-stage process:

(i) Data import and initial screening: all identified studies were imported from a standardized data extraction form to capture data relevant to the research questions and to remove duplicates. After de-duplication, titles and abstracts underwent initial screening independently. The selection process adhered to the PRISMA guidelines (Figure 1).

(ii) Data charting and synthesis: Data from the included studies were extracted into a data charting form, capturing the following variables, year of publication, study design, data source, vaccine doses, primary study objectives, overall VSC rates, series completion rates for different vaccines (if applicable), and common predictors such as knowledge of immunization and maternal education; education level of caregiver; household wealth status; place of delivery; number of antenatal or postnatal care visits; possession of a vaccination card; place of residence; distance to a healthy facility or travel time; number of children under five in the household; and the gender of the head household or decision maker.

Extracted data were organized into a data charting form to facilitate synthesis. A narrative synthesis and interpretation of the data were then undertaken. The characteristics of included studies were summarized using tables, charts, and maps for enhanced visualization. Evidence on VSC rates was summarized by key study objectives, including (i) prevalence of VSC in SSA; (ii) trends of VSC in SSA; and (iii) inequality of VSC in SSA.

## 3. Results

### 3.1. Overview of Included Studies

Searches across five databases (PubMed, Embase, CINAHL, Web of Science, and Google Scholar) retrieved a total of 9503 articles. Of these, 53 met the inclusion/exclusion criteria and passed the quality assessment [12,13,14,15,16,17,18,19,20,21,22,23,24,25,26,27,28,29,30,31,32,33,34,35,36,37,38,39,40,41,42,43,44,45,46,47,48,49,50,51,52,53,54,55,56,57,58,59,60,61,62,63,64].

Study design and data sources: As summarized in Table 1, the majority of included studies were cross-sectional (32/53, 60.4%), followed by secondary data analyses (18/53, 34.0%), cohort studies (2/53, 3.8%), and randomized controlled trials (RCTs) (1/53, 1.9%). Most secondary data analyses utilized data from DHSs at national or regional levels, except for two studies that used data from the Multiple Indicator Cluster Surveys (MICSs) [48,54]. The remaining 36 studies relied on primary data sources, including structured questionnaires, WHO EPI cluster surveys [38], and interviewer-administered data collection instruments [22]. Among studies using DHS or MICS data, the publication delay (defined as the median of time difference between the study year and publication year) was 4.0 (3.0, 6.0) years. In contrast, primary studies had a median delay of 2.0 (1.0, 3.0) years from implementation to publication.

### 3.2. Study Settings

**Figure 2:**
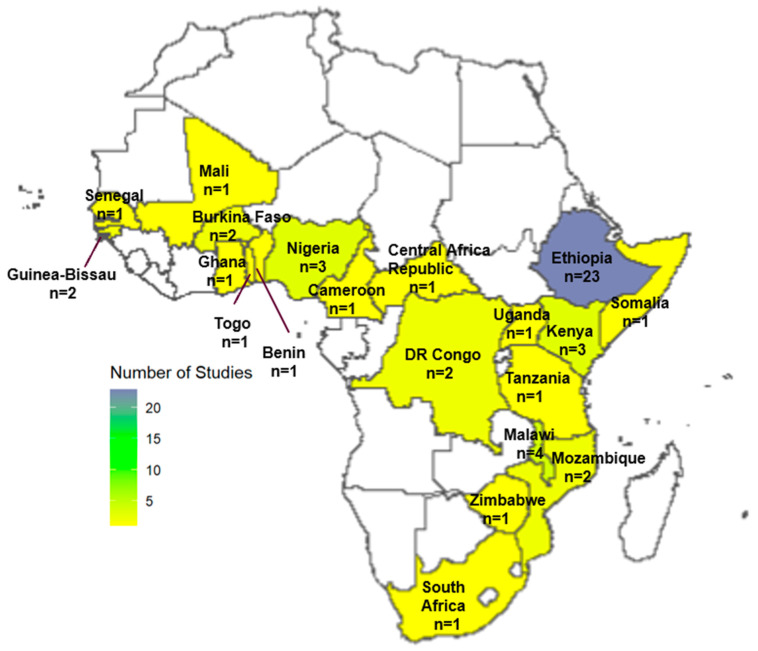
Spatial distribution of included studies.

Of the 53 included studies, the majority (*n* = 35, 66.0%) were sub-national, focusing on specific regions within countries, while 18 studies were conducted at the national level. A significant proportion of studies were conducted in Ethiopia (*n* = 23, 43.4%), followed by Malawi (*n* = 4, 7.5%), Nigeria (*n* = 3, 5.7%), and Kenya (*n* = 3, 5.7%). Figure 2 illustrates the geographical distribution of the included studies by country.

### 3.3. Vaccines Assessed in the Published Literature

**Figure 3:**
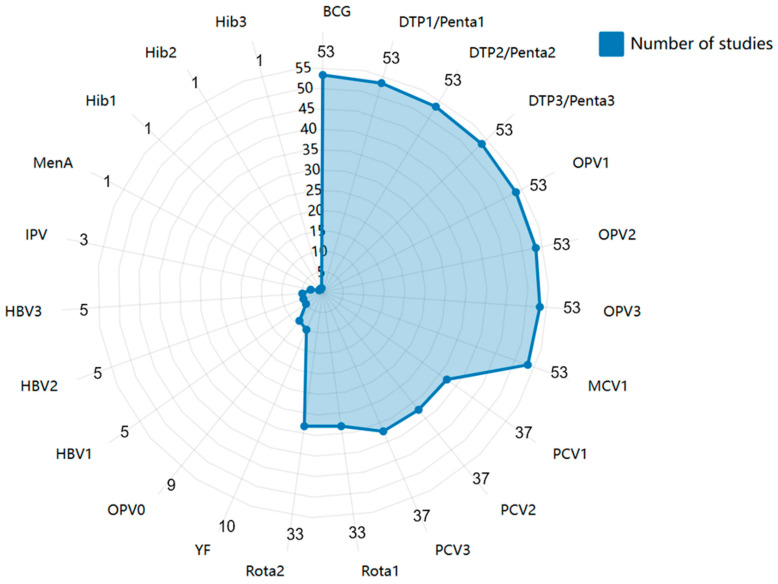
Vaccines assessed in the published literature. Abbreviations: BCG, Bacillus Calmette-Guérin Vaccine; DTP1/2/3, the first/second/third doses of diphtheria, tetanus, and pertussis vaccine, similarly below; OPV, oral poliovirus vaccine; MCV, measles-containing vaccine; PCV, pneumococcal conjugate vaccine; Rota, rotavirus vaccine; YF, Yellow Fever vaccine; HBV, Hepatitis B Vaccine; MenA, Meningococcal A vaccine; Hib, Haemophilus influence type b vaccine.

The most commonly assessed vaccines for VSC among children aged 12–23 months in SSA are BCG, DTP/Penta (first, second, and third doses), OPV (first, second, and third doses), and MCV1. In contrast, Meningococcal A (MenA) and Haemophilus influenzae type b (Hib) were the least studied antigens. Only nine studies (17.0%) included the birth dose of OPV (OPV0) in their VSC measurement, despite all 20 countries recommending OPV0 in their National Immunization Programs (NIPs) (Figure 3).

#### 3.3.1. Prevalence of VSC in SSA

The overall VSC and series completion rates for the six most commonly included vaccines in NIPs of SSA countries are summarized in Supplementary Section S3. Among the 53 studies reviewed, 26 (49.1%) focused primarily on the prevalence of VSC among children aged 12–23 months, with 18 reporting national-level prevalence data. VSC rates varied widely, ranging from 36.6% in Burkina Faso to 95.2% in Ghana.

#### 3.3.2. Trends of VSC in SSA

Only three studies (5.7%) explored trends in VSC among children aged 12–23 months. In Ghana, a secondary analysis of DHS data between 1998 and 2004 showed an increasing trend in VSC, with rates rising from 85.2% in 1998 to 95.2% in 2014 [53]. In Malawi, a bottleneck analysis of DHS data revealed an increase in VSC from 65% in 2004 to 84% in 2010, followed by a decline to 73% between 2015 and 2016. Conversely, in Tanzania, a cross-sectional study comparing vaccination data from rural area in 1998–1999 and 2006–2007 reported a declining VSC rate from 71.6% in 1998 to 57.2% in 2007 [62].

#### 3.3.3. Inequality of VSC in SSA

Few studies (*n* = 4, 7.5%) examined inequalities in VSC among children aged 12–23 months. Two studies investigated rural–urban disparities in Ethiopia. Luman et al. [34] reported a VSC rate of 16.1% in the urban Ambo district compared to 3.7% in the rural Yaya-Gulelena D/Libanos district in 2003. Similarly, Asmare et al. [17] found VSC rates of 66.1% in urban areas versus 59.2% in rural southwest Ethiopia. In Nigeria, Fatiregun et al. [39] observed minimal differences in VSC rates between urban (41.3%) and rural (40.2%) districts. A secondary analysis of Kenya’s 2014 DHS data revealed regional disparities, with VSC rates ranging from 42% in the northeastern region to 78% in the central and eastern regions.

#### 3.3.4. Factors Associated with VSC in SSA

Among the 43 studies (81.1%) examining factors associated factors with VSC, the most commonly reported predictors were as follows:

Individual-level predictors: (i) caregiver education level (22/43, 51.2%); (ii) household wealth status (19/43, 44.2%); (iii) number of children under five in the household (19/43, 44.2%); (iv) knowledge of immunization and maternal education (18/43, 41.9%); (v) place of residence (18/43, 41.9%); and (vi) gender of the household head or decision maker (10/43, 23.3%). Community-level predictors: (i) utilization of antenatal or postnatal care visits (19/43, 44.2%); (ii) place of delivery (19/43, 44.2%); (iii) distance to a health facility or travel time (14/43, 32.6%); and (iv) possession of a vaccination card (5/43, 11.6%).

**Figure 4:**
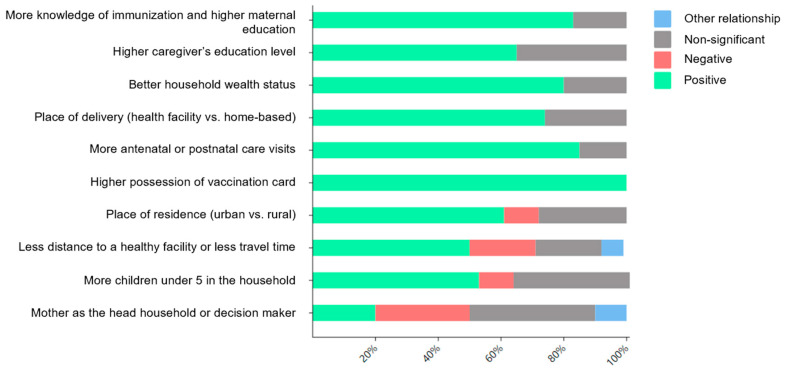
Most commonly reported predictors of vaccine series completion used in the published literature.

Figure 4 illustrates the relationship between these factors and VSC. Higher VSC rates were most strongly associated with (i) greater knowledge of immunization and maternal education (15/18, 83.3%); (ii) higher caregiver education level (14/22, 63.6%); (iii) better household wealth status (15/19, 78.9%); (iv) health facility-based deliveries (14/19, 73.7%); (v) increased antenatal/postnatal care visits (16/19, 84.2%); (vi) possession of a vaccination card (5/5, 100%); urban residence (11/18, 61.1%); (vii) distance to a healthy facility or travel time (7/14, 50%); and (viii) number of children under five in the household (10/19, 52.63%).

No clear relationship was observed for the gender of the household head or decision maker, with mixed findings across studies: 20% (2/10) reported a positive relationship, 30% (3/10) reported a negative relationship, 40% (4/10) reported a non-significant relationship, and 10% (1/10) were of another relationship (both mother and father made decisions).

## 4. Discussion

This scoping review synthesizes existing evidence on VSC among children aged 12–23 months in SSA, focusing on study methodologies, explored domains, and associated factors. A total of 53 studies from 20 SSA countries (2000–2023) were included, with most using cross-sectional designs based on population surveys. Prevalence and associated factors were the most commonly studied domains, while less attention was given to inequalities and trends. Ten key factors associated with VSC included caregiver education, household wealth, number of children, immunization knowledge, maternal education, place of residence, and access to healthcare services. This review highlights critical research gaps and areas for further investigation.

The majority of studies (96.2%) utilized cross-sectional analyses, using either secondary data from DHS or primary data collected through DHS-adapted questionnaires. This underscores the importance of population-based surveys in evaluating country-specific immunization programs in SSA. However, DHS data are not universally available in all low- and middle-income countries (LMICs) and are conducted at irregular intervals [65]. Primary cross-sectional studies could complement secondary data by offering a more current reflection of population immunization status.

Notably, almost half of the studies (43.4%) were conducted in Ethiopia, while fewer than five studies originated from each of the remaining 19 countries. It is noteworthy that numerous surveys were excluded from our review due to common methodological limitations. A significant number did not meet the inclusion criteria, primarily due to methodological inconsistencies, incomplete reporting of VSC rates, and absence of standardized definitions aligned with immunization schedules. Many studies also lacked sufficient disaggregation of data by demographic and geographic factors, hindering the assessment of vaccination coverage inequalities. Adhering to robust methodological standards is essential for generating reliable data to inform immunization policies and interventions. These gaps highlight critical areas for improvement in immunization coverage surveys across SSA. Standardized approaches to measuring and reporting complete vaccination series data for specific age cohorts would significantly enhance the quality of evidence available to policy-makers and program implementers. Future research should prioritize standardized definitions, comprehensive data collection, and transparent reporting to enhance the evidence base on VSC in SSA.

This imbalance highlights a lack of evidence from over half of SSA countries. Given the diversity of immunization schedules and contextual factors influencing coverage, more country-specific data are essential to inform effective policy-making.

The absence of studies meeting our inclusion criteria from several countries in SSA is concerning, particularly in Western African nations and those with high HIV prevalence rates, such as Namibia, Botswana, Zimbabwe, and Zambia [66]. This gap raises important questions about vaccination coverage monitoring in these regions. The lack of data limits our ability to assess vaccination coverage in populations that may be more vulnerable to infectious diseases due to immunocompromised conditions. While national immunization programs exist in these countries, the absence of studies may reflect gaps in data collection and reporting rather than actual immunization coverage. Future research should prioritize these underrepresented countries to ensure a comprehensive understanding of VSC across SSA. Additionally, further investigation is needed to determine whether immunization rates in these countries are indeed lower than in regions where studies have been conducted. This study synthesized on the prevalence of VSC among children aged 12–23 months across SSA. However, it identified significant gaps in the published literature on trends and inequalities in VSC. Among the 20 countries covered in this study, only Ghana achieved VSC prevalence rates exceeding 90%, consistent with a 2019 WHO report estimating that 40/46 SSA countries require substantial improvements in vaccination coverage to meet the GVAP goals [65]. Yet, a secondary analysis by Donfouet et al. found that the series completion rate for basic childhood vaccinations (BCG, Penta, polio, and MCV) in Ghana was only 79.4% in 2008 and 78.2% in 2014 [67]. Due to inconsistencies in VSC reporting, varying contextual factors, and diverse immunization schedules across studies, it was not possible to calculate an overall VSC rate or identify general trends in SSA. Only three studies documented trends in VSC across several SSA countries over the past two decades.

Between 2000 and 2023, Ethiopia’s national VSC rate increased from 24.6% to 57.8%, while sub-national rates rose from 3.7% to 77.2%. This aligns with research by Yibeltal et al. [68], which showed a steady increase in Ethiopia’s VSC rates, rising from 14.3% in 2000 to 44.1% in 2019, driven by improved immunization planning at both national and sub-national levels, strengthened financial resources, and enhanced health service delivery. Similarly, Malawi demonstrated steady growth in VSC rates from 2005 to 2015, although a slight decline occurred between 2015 and 2016. This decline may be explained by the introduction of the second dose of MCV in 2015 and the transition from trivalent OPV to bivalent OPV in 2016, as Fisker et al. noted that changes in immunization practices and the introduction of new vaccines can negatively impact vaccination coverage [51,69].

Our review identified a gap in research examining oral polio vaccine [OPV] series completion among children in SSA. Despite the ongoing occurrence of polio cases in parts of SSA, studies assessing complete OPV series coverage were notably absent from our findings. This research gap is particularly concerning given that polio causes devastating, lifelong sequelae even when not fatal, yet is entirely preventable through complete full vaccination. The persistence of polio in certain regions may be directly linked to incomplete vaccine series administration or coverage gaps [70]. We strongly recommend that future research investigate OPV series completion rates, barriers to completion, and targeted interventions to ensure full protection. Such research is critical to informing eradication efforts and preventing unnecessary suffering from this preventable disease, particularly in regions where cases continue to be reported despite global eradication efforts. Strengthening surveillance and reporting mechanisms for polio vaccination series completion should be prioritized alongside other routine immunizations.

Evidence from the literature also highlights inequalities in VSC within SSA countries, particularly between urban and rural areas. These findings align with Bobo et al., who reported pro-urban disparities in full vaccination coverage across 23 SSA countries [71]. Similarly, Ameyaw et al. emphasized rural–urban disparities, noting that children in urban settings were more likely to be fully immunized than those in rural areas [72].

This review revealed substantial within-country disparities in immunization coverage, with pronounced regional variations. These geographical disparities likely stem from multiple interconnected factors. Limited transportation infrastructure appears to be a significant barrier, particularly in rural and remote areas, contributing to the urban advantage in vaccination coverage. Transportation challenges intersect with other factors, including healthcare facility distribution, vaccine supply chain reliability, and healthcare workforce shortages in underserved areas [73]. Interventions such as Burkina Faso’s outreach programs demonstrate promising approaches to addressing these disparities. These programs deliver vaccination services directly to communities through mobile clinics and community health workers, effectively bypassing transportation barriers. We recommend expanding such outreach initiatives across SSA with context-specific adaptations. However, implementation challenges include securing sustainable funding, maintaining cold chain during transport, training and retaining community health workers, and integrating these programs into existing health systems. Context-specific solutions must address regional infrastructure limitations while leveraging local community structures to enhance vaccination accessibility in hard-to-reach areas. Furthermore, a cross-sectional study conducted across 35 countries SSA identified factors associated with these disparities, including access to health facilities, household wealth, socioeconomic status, and maternal education level [74].

The role of governmental policies and governance structures in shaping vaccination outcomes is critical. In some SSA countries, national immunization programs have been strengthened through policy commitments, funding allocations, and international partnerships [75]. However, political instability, inadequate resource allocation, and inconsistent policy implementation remain significant barriers to achieving high VSC rates [76]. Countries with strong national immunization frameworks and sustained government commitment have demonstrated better vaccination outcomes. For example, proactive policy approaches, such as integrating immunization services with maternal and child health programs, have improved VSC in certain regions. Addressing these governance challenges requires sustained political will, increased investments in health infrastructure, and continuous monitoring to ensure policies effectively translate into improved immunization coverage.

Factors influencing VSC were commonly assessed, with notable variations across studies and settings. Positive factors included knowledge of immunization, maternal education, caregiver education level, and household wealth in LMICs. A systematic review conducted in LMICs revealed that low VSC rates in children are largely attributable to a lack of maternal education, unawareness of immunization benefits, and mistrust in the safety and efficacy of vaccines [77]. Additionally, parents with lower education level and socioeconomic status demonstrated greater uncertainty toward immunization [78].

The positive association between place of residence and VSC was reported in over half of the literature (61.1%). Children born in urban areas generally have better access to vaccination services and greater utilization of health facilities compared to those in rural regions. However, a study conducted in Burkina Faso reported contrasting results, showing a significantly higher prevalence of VSC among children aged 12–23 months in rural areas than in urban settings [79]. This finding was attributed to the presence of outreach vaccination teams in rural areas, whereas children in urban areas relied on accessing vaccination services through health facilities.

The findings of this scoping review reveal significant gaps in VSC among children aged 12–23 months in SSA, a situation requiring urgent attention and action. While most studies in this review focused on identifying barriers to vaccination completion, a shift toward holistic immunization approaches is needed. As demonstrated by El-Halabi et al. (2023) in Jordan’s Zaatari refugee camp and Ngah et al. (2023) in rural Cameroon, effective vaccination serves not only as disease prevention but also as a long-term investment in children’s health, which in turn influences the public health characteristics of future societies [80,81].

To improve VSC rates in SSA, we recommend implementing targeted digital reminder systems, developing community-based programs that address social determinants of vaccination completion, integrating vaccination services with other routine child health services, and investing in health worker training to enhance communication on the long-term societal benefits of complete immunization for caregivers. These approaches acknowledge the gravity of incomplete vaccination coverage while providing concrete pathways toward ensuring that children throughout SSA receive full immunization protection.

This scoping review was the first to identify methodological gaps in the literature on VSC among children aged 12–23 months across 21 SSA countries. A systematic and comprehensive search strategy was employed, ensuring the inclusion of a wide range of topics related to childhood vaccination coverage in SSA. This approach minimized the risk of missing relevant studies due to variations in terminology. The review revealed that VSC rates were often linked to the timeliness of vaccination and immunization drop-out rates.

However, this study has some limitations. The gray literature from sources such as the WHO and national health ministries was not included. Additionally, the search was restricted to five databases and limited to studies published in English, which may affect the generalizability of the findings. Lastly, nearly half of the included studies were conducted in Ethiopia, potentially limiting the generalizability of the findings to the broader SSA region. The high representation of Ethiopia in the literature may be due to greater data availability, more frequent health surveys, or a stronger research focus on immunization programs. However, it is essential to acknowledge that vaccination challenges, health system structures, and sociopolitical factors vary across SSA countries. Future research should aim for more balanced geographic representation.

## 5. Conclusions

This scoping review identified 53 studies examining VSC among children aged 12–23 months across 20 SSA countries. Most studies utilized cross-sectional analyses of population-based surveys, with primary studies exhibiting less publication delay compared to secondary analyses. The prevalence and associated factors of VSC were the most frequently explored domains, whereas limited evidence was found on inequalities and trends in VSC. Key factors with VSC among children in SSA were identified, although patterns varied across studies and settings. Future research should address the methodological gaps highlighted in this review, particularly regarding inequalities and trends in VSC. Sustained efforts through policy-making and evidence-based interventions are essential to achieve optimal VSC prevalence in SSA and improve immunization outcomes.

## Figures and Tables

**Table 1:** Summarized characteristics of included studies.

Characteristics	N (%)
Publication year	
2000–2010	4 (7.5%)
2011–2023	49 (92.5%)
Publication delay ^†^	
Secondary analysis	4.0 (3.0, 6.0) years
Primary studies	2.0 (1.0, 3.0) years
Study design	
Cross-sectional	31 (60.4%)
Secondary analysis	19 (34.0%)
Cohort	2 (3.8%)
Randomized control trial	1 (1.9%)
Data sources	
Demographic Health Survey	29 (54.7%)
Multiple Indicator Cluster Survey	2 (3.8%)
Primary data collection	22 (41.5%)
Primary objective of study	
Prevalence	26 (49.1%)
Inequalities	4 (7.5%)
Trends	3 (5.7%)
Associated factors	43 (81.1%)
Others	8 (15.1%)

^†^ Publication delay was described as median (interquartile range, IQR).

## Data Availability

The original contributions presented in this study are included in the article/Supplementary Material. Further inquiries can be directed to the corresponding author.

## Supplementary Materials

The following supporting information can be downloaded at https://www.mdpi.com/article/10.3390/children12040415/s1; Supplementary Section S1: PRISMA-ScR Checklist; Supplementary Section S2: Database search terms and results; Supplementary Section S3: Characteristics and key findings of included studies.
