# Supplementary material for: Characteristics of Studies Focusing on Vaccine Series Completion Among Children Aged 12–23 Months in Sub-Saharan Africa: A Scoping Review
Source: Children (Basel). 2025 Mar 26;12(4):415. doi: 10.3390/children12040415 (PMC12025459; doi:10.3390/children12040415)
Supplement: Supplementary file 1 [file children-12-00415-s001.zip › children-3488649-supplementary.pdf]

Supplementary Materials

Section S1. Preferred Reporting Items for Systematic reviews and Meta-Analyses extension for Scoping Reviews (PRISMA-ScR) Checklist

Title: Characteristics of Studies Focusing on Vaccine Series Completion Among Children Aged 12–23 Months in Sub-Saharan Africa: A Scoping Review

SECTION	ITEM	PRISMA-ScR CHECKLIST ITEM	REPORTED ON PAGE #
**TITLE**			
Title	1	Identify the report as a scoping review.	Page 1
**ABSTRACT**			
Structured summary	2	Provide a structured summary that includes (as applicable): background, objectives, eligibility criteria, sources of evidence, charting methods, results, and conclusions that relate to the review questions and objectives.	Page 1
**INTRODUCTION**			
Rationale	3	Describe the rationale for the review in the context of what is already known. Explain why the review questions/objectives lend themselves to a scoping review approach.	Page 2
Objectives	4	Provide an explicit statement of the questions and objectives being addressed with reference to their key elements (e.g., population or participants, concepts, and context) or other relevant key elements used to conceptualize the review questions and/or objectives.	Page 3
**METHODS**			
Protocol and registration	5	Indicate whether a review protocol exists; state if and where it can be accessed (e.g., a Web address); and if available, provide registration information, including the registration number.	Page 3
Eligibility criteria	6	Specify characteristics of the sources of evidence used as eligibility criteria (e.g., years considered, language, and publication status), and provide a rationale.	Page 3
Information sources*	7	Describe all information sources in the search (e.g., databases with dates of coverage and contact with authors to identify additional sources), as well as the date the most recent search was executed.	Page 3
Search	8	Present the full electronic search strategy for at least 1 database, including any limits used, such that it could be repeated.	Page 3, and appendix 2
Selection of sources of evidence†	9	State the process for selecting sources of evidence (i.e., screening and eligibility) included in the scoping review.	Page 4
Data charting process‡	10	Describe the methods of charting data from the included sources of evidence (e.g., calibrated	Page 4

SECTION	ITEM	PRISMA-ScR CHECKLIST ITEM	REPORTED ON PAGE #
		forms or forms that have been tested by the team before their use, and whether data charting was done independently or in duplicate) and any processes for obtaining and confirming data from investigators.	
Data items	11	List and define all variables for which data were sought and any assumptions and simplifications made.	Page 4 and appendix 3
Critical appraisal of individual sources of evidence§	12	If done, provide a rationale for conducting a critical appraisal of included sources of evidence; describe the methods used and how this information was used in any data synthesis (if appropriate).	Not done because not explicitly required for a scoping review
Synthesis of results	13	Describe the methods of handling and summarizing the data that were charted.	Page 5
**RESULTS**			
Selection of sources of evidence	14	Give numbers of sources of evidence screened, assessed for eligibility, and included in the review, with reasons for exclusions at each stage, ideally using a flow diagram.	Page 6
Characteristics of sources of evidence	15	For each source of evidence, present characteristics for which data were charted and provide the citations.	Page 7
Critical appraisal within sources of evidence	16	If done, present data on critical appraisal of included sources of evidence (see item 12).	Not done because not explicitly required for a scoping review
Results of individual sources of evidence	17	For each included source of evidence, present the relevant data that were charted that relate to the review questions and objectives.	Page 6 -12
Synthesis of results	18	Summarize and/or present the charting results as they relate to the review questions and objectives.	Page 6 -12
**DISCUSSION**			
Summary of evidence	19	Summarize the main results (including an overview of concepts, themes, and types of evidence available), link to the review questions and objectives, and consider the relevance to key groups.	Page 12 - 16
Limitations	20	Discuss the limitations of the scoping review process.	Page 16
Conclusions	21	Provide a general interpretation of the results with respect to the review questions and objectives, as well as potential implications and/or next steps.	Page 17
**FUNDING**			
Funding	22	Describe sources of funding for the included sources of evidence, as well as sources of funding for the scoping review. Describe the role of the funders of the scoping review.	Not applicable

JBI = Joanna Briggs Institute; PRISMA-ScR = Preferred Reporting Items for Systematic reviews and Meta-Analyses extension for Scoping Reviews.
* Where *sources of evidence* (see second footnote) are compiled from, such as bibliographic databases, social media platforms, and Web sites.

† A more inclusive/heterogeneous term used to account for the different types of evidence or data sources (e.g., quantitative and/or qualitative research, expert opinion, and policy documents) that may be eligible in a scoping review as opposed to only studies. This is not to be confused with *information sources* (see first footnote).
‡ The frameworks by Arksey and O'Malley (6) and Levac and colleagues (7) and the JBI guidance (4, 5) refer to the process of data extraction in a scoping review as data charting.
§ The process of systematically examining research evidence to assess its validity, results, and relevance before using it to inform a decision. This term is used for items 12 and 19 instead of "risk of bias" (which is more applicable to systematic reviews of interventions) to include and acknowledge the various sources of evidence that may be used in a scoping review (e.g., quantitative and/or qualitative research, expert opinion, and policy document).

From: Tricco AC, Lillie E, Zarin W, O'Brien KK, Colquhoun H, Levac D, et al. PRISMA Extension for Scoping Reviews (PRISMAScR): Checklist and Explanation. Ann Intern Med. 2018;169:467–473. doi: 10.7326/M18-0850.

Section. S2 Database search terms and results

1.PubMed 2024.01.25

Key words	Searches	Results
#1 (Vaccination)	(vaccine[MeSH Terms]) OR (vaccination[MeSH Terms])) OR (immunization[MeSH Terms])) OR (vaccine[Text Word])) OR (vaccination[Text Word])) OR (immunization[Text Word])	492,249
#2 (Coverage)	(coverage[Text Word]) OR (completion[Text Word])) OR (uptake[Text Word])) OR (rate[Text Word])) OR (proportion[Text Word])	3,688,048
#3 (Children)	(children[Title/Abstract]) OR (childhood[Title/Abstract])) OR (infant[Title/Abstract])) OR (toddler[Title/Abstract])) OR (under 2 years[Title/Abstract])) OR (12-23 months[Title/Abstract])	1,435,647
#4 (Sub-Saharan Africa)	((Sub Saharan Africa) OR (Angola]) OR (Benin) OR (Botswana) OR (Burkina Faso) OR (Burundi) OR (Cabo Verde) OR (Cameroon) OR (Central African Republic) OR (Chad) OR (Comoros) OR (Congo) OR (Democratic Republic Congo) OR (Cote d'Ivoire) OR (Ivory Coast) OR (Djibouti) OR (Equatorial Guinea) OR (Eritrea) OR (Ethiopia) OR (Gabon) OR (Gambia) OR (Ghana) OR (Guinea) OR (Guinea-Bissau) OR (Kenya) OR (Lesotho) OR (Liberia) OR (Madagascar) OR (Malawi) OR (Mali) OR (Mauritania) OR (Mauritius) OR (Mozambique) OR (Namibia) OR (Niger) OR (Nigeria) OR (Reunion) OR (Rwanda) OR (Sao Tome Principe) OR (Senegal) OR (Seychelles) OR (Sierra Leone) OR (Somalia) OR (South Africa) OR (Sudan) OR (Swaziland) OR (Tanzania) OR (Togo) OR (Uganda) OR (Zambia) OR (Zimbabwe) OR (East Africa) OR (Middle Africa) OR (Southern Africa) OR (West Africa) OR (Central Africa) OR (Western Sahara))	700,022
#5	#1 AND #2 AND #3 AND #4	3,270
#6	2000-2023 (Publication Years) and English (Languages)	2,675

2. Embase 2024.01.25

Key words	Searches	Results
#1 (Vaccination)	'vaccines'/exp OR vaccines OR vaccinations OR immunizations	535,803
#2 (Coverage)	'coverage'/exp OR coverage OR uptake OR 'completion'/exp OR completion OR rate OR proportion	5,804,477
#3 (Children)	children:ab,ti OR childhood:ab,ti OR infant:ab,ti OR toddler:ab,ti OR 'under 2 years':ab,ti OR '12-23 months':ab,ti	2,142,458
#4 (Sub-Saharan Africa)	'sub saharan africa':ab,ti OR angola:ab,ti OR benin:ab,ti OR botswana:ab,ti OR 'burkina faso':ab,ti OR burundi:ab,ti OR 'cabo verde':ab,ti OR cameroon:ab,ti OR 'central african republic':ab,ti OR chad:ab,ti OR comoros:ab,ti OR congo:ab,ti OR 'democratic republic congo':ab,ti OR 'cote d ivoire':ab,ti OR 'ivory coast':ab,ti OR djibouti:ab,ti OR 'equatorial guinea':ab,ti OR eritrea:ab,ti OR ethiopia:ab,ti OR gabon:ab,ti OR gambia:ab,ti OR ghana:ab,ti OR guinea:ab,ti OR 'guinea bissau':ab,ti OR kenya:ab,ti OR lesotho:ab,ti OR liberia:ab,ti OR madagascar:ab,ti OR malawi:ab,ti OR mali:ab,ti OR mauritania:ab,ti	498,850

	OR mauritius:ab,ti OR mozambique:ab,ti OR namibia:ab,ti OR niger:ab,ti OR nigeria:ab,ti OR reunion:ab,ti OR rwanda:ab,ti OR 'sao tome principe':ab,ti OR senegal:ab,ti OR seychelles:ab,ti OR 'sierra leone':ab,ti OR somalia:ab,ti OR 'south africa':ab,ti OR sudan:ab,ti OR swaziland:ab,ti OR tanzania:ab,ti OR togo:ab,ti OR uganda:ab,ti OR zambia:ab,ti OR zimbabwe:ab,ti OR 'east africa':ab,ti OR 'middle africa':ab,ti OR 'southern africa':ab,ti OR 'west africa':ab,ti OR 'central africa':ab,ti OR 'western sahara':ab,ti	
#5	1 AND #2 AND #3 AND #4	2,531
#6 Filters	2000-2023 (Publication Years) and English (Languages) and Preschool (age)	2,203

3. CINAHL 2024.01.26

Key words	Searches	Results
#1 (Vaccine coverage)	TX (vaccines or vaccinations or immunizations) AND TX (coverage or uptake or completion or rate or proportion)	26,969
#2 (children)	TI (children or childhood or (under 2 years) or (12-23 months) or infant or toddler) OR AB (children or childhood or (under 2 years) or (12-23 months) or infant or toddler)	662,504
#3 (Sub-Saharan Africa)	TI ((Sub Saharan Africa) OR (Angola) OR (Benin) OR (Botswana) OR (Burkina Faso) OR (Burundi) OR (Cabo Verde) OR (Cameroon) OR (Central African Republic) OR (Chad) OR (Comoros) OR (Congo) OR (Democratic Republic Congo) OR (Cote d'Ivoire) OR (Ivory Coast) OR (Djibouti) OR (Equatorial Guinea) OR (Eritrea) OR (Ethiopia) OR (Gabon) OR (Gambia) OR (Ghana) OR (Guinea) OR (Guinea-Bissau) OR (Kenya) OR (Lesotho) OR (Liberia) OR (Madagascar) OR (Malawi) OR (Mali) OR (Mauritania) OR (Mauritius) OR (Mozambique) OR (Namibia) OR (Niger) OR (Nigeria) OR (Reunion) OR (Rwanda) OR (Sao Tome Principe) OR (Senegal) OR (Seychelles) OR (Sierra Leone) OR (Somalia) OR (South Africa) OR (Sudan) OR (Swaziland) OR (Tanzania) OR (Togo) OR (Uganda) OR (Zambia) OR (Zimbabwe) OR (East Africa) OR (Middle Africa) OR (Southern Africa) OR (West Africa) OR (Central Africa) OR (Western Sahara))	88,629
#3	#1 AND #2 AND #3	985
#4 Filters	2000-2023 (Publication Years) and English (Languages)	964

4.Web of Science 2024.01.26

Key words	Searches	Results
#1 (Childhood Vaccination)	(vaccine OR vaccination OR immunization (All Fields)) AND (coverage OR uptake OR completion OR rate OR proportion (All Fields)) AND (children OR childhood OR infant OR toddler OR (under 2 years) OR (12-23 months) (Topic)) AND ((Sub Saharan Africa) OR (Angola]) OR (Benin) OR (Botswana) OR (Burkina Faso) OR (Burundi) OR (Cabo Verde) OR (Cameroon) OR (Central African Republic) OR (Chad) OR (Comoros) OR (Congo) OR (Democratic Republic Congo) OR (Cote d'Ivoire) OR (Ivory Coast) OR (Djibouti) OR (Equatorial Guinea) OR (Eritrea) OR (Ethiopia) OR (Gabon) OR (Gambia) OR (Ghana) OR (Guinea) OR (Guinea-Bissau) OR (Kenya) OR (Lesotho) OR (Liberia) OR	4,121

	(Madagascar) OR (Malawi) OR (Mali) OR (Mauritania) OR (Mauritius) OR (Mozambique) OR (Namibia) OR (Niger) OR (Nigeria) OR (Reunion) OR (Rwanda) OR (Sao Tome Principe) OR (Senegal) OR (Seychelles) OR (Sierra Leone) OR (Somalia) OR (South Africa) OR (Sudan) OR (Swaziland) OR (Tanzania) OR (Togo) OR (Uganda) OR (Zambia) OR (Zimbabwe) OR (East Africa) OR (Middle Africa) OR (Southern Africa) OR (West Africa) OR (Central Africa) OR (Western Sahara) (Topic)	
#4 Filters	2000-2023 (Publication Years) and English (Languages)	3,661

5.Google Scholar 2024.03.26

Key words	Searches	Results
#1 Search terms	(vaccine OR vaccination OR immunization) AND (coverage OR uptake OR completion OR rate OR proportion) AND (children OR childhood OR infant OR toddler OR (under 2 years) OR (12-23 months)) AND ((Sub Saharan Africa) OR (Angola]) OR (Benin) OR (Botswana) OR (Burkina Faso) OR (Burundi) OR (Cabo Verde) OR (Cameroon) OR (Central African Republic) OR (Chad) OR (Comoros) OR (Congo) OR (Democratic Republic Congo) OR (Cote d'Ivoire) OR (Ivory Coast) OR (Djibouti) OR (Equatorial Guinea) OR (Eritrea) OR (Ethiopia) OR (Gabon) OR (Gambia) OR (Ghana) OR (Guinea) OR (Guinea-Bissau) OR (Kenya) OR (Lesotho) OR (Liberia) OR (Madagascar) OR (Malawi) OR (Mali) OR (Mauritania) OR (Mauritius) OR (Mozambique) OR (Namibia) OR (Niger) OR (Nigeria) OR (Reunion) OR (Rwanda) OR (Sao Tome Principe) OR (Senegal) OR (Seychelles) OR (Sierra Leone) OR (Somalia) OR (South Africa) OR (Sudan) OR (Swaziland) OR (Tanzania) OR (Togo) OR (Uganda) OR (Zambia) OR (Zimbabwe) OR (East Africa) OR (Middle Africa) OR (Southern Africa) OR (West Africa) OR (Central Africa) OR (Western Sahara)	16400
#2 Filters	2000-2023 (Publication Years) and English (Languages) and first 200 results	200
	Number of selected results that met the eligibility criteria from first 200 search results	8

Section S3. Characteristics and key findings of included studies

Table S1. Characteristics of the included studies

Commented [M1]: We renumbered the table. Please confirm.

**Author & date**	**Place of study**	**Study Design**	**Data Sources**	**Sample size**	**Vaccines assessed in the published literature**
Sako et al., 2023 (12)	Ethiopia	Secondary analysis	DHS 2019	1,008	BCG+3Penta+3OPV +3PCV+2RV+MCV
Gelagay et al., 2023 (13)	Dabat district, Ethiopia	Secondary analysis	DHS 2020	857	BCG+3Penta+3OPV +3PCV+2RV+MCV
Worku et al., 2022 (14)	Ethiopia	Cross-sectional	Questionnaire in 2020	3,016	BCG+3Penta+3OPV +3PCV+2RV+MCV
Muluye et al., 2022 (15)	Haramaya district, Easten, Ethiopia	Cross-sectional	Questionnaire in 2021	892	BCG+3Penta+4OPV +3PCV+2RV+MCV
Darebo et al., 2022 (16)	Demba Gofa District, Southern, Ethiopia	Cross-sectional	Questionnaire in 2019	677	BCG+3Penta+3OPV +3PCV+2RV+MCV
Asmare et al., 2022 (17)	Southwest, Ethiopia	Cross-sectional	Questionnaire in 2021	644	BCG+3Penta+3OPV +3PCV+2RV+MCV
Mekonnen et al., 2021 (18)	Gonder city, northwest, Ethiopia	RCT in 2020		434	BCG+3Penta+3OPV +3PCV+2RV+MCV
Miretu et al., 2021 (19)	Dessie town, Northeast, Ethiopia	Cross-sectional	Questionnaire in 2020	633	BCG+3Penta+3OPV +3PCV+2RV+MCV
Jimma et al., 2021 (20)	Assosa Town, Western, Ethiopia	Cross-sectional	Questionnaire in 2020	372	BCG+3Penta+4OPV +3PCV+2RV+MCV+ IPV

Author & date	Place of study	Study Design	Data Sources	Sample size	Vaccines assessed in the published literature
Gelagay et al., 2021 (21)	Wogera district, Northwest, Ethiopia	Cross-sectional	Questionnaire in 2020	584	BCG+3Penta+3OPV +3PCV+2RV+MCV
Mekonnen et al., 2020 (22)	Gondar city, Northwest, Ethiopia	Cross-sectional	Interviewer-administered data collection instrument in 2018	774	BCG+3Penta+3OPV +3PCV+2RV+MCV+ IPV
Geweniger et al., 2020 (23)	Ethiopia	Secondary analysis	DHS 2016	2,004	BCG+3Penta+3OPV +3PCV+2RV+MCV
Debie et al., 2020 (24)	Ethiopia	Secondary analysis	DHS 2011 and 2016	3,791	BCG+3Penta+3OPV +3PCV+2RV+MCV
Debie et al., 2020 (25)	Ethiopia	Secondary analysis	DHS 2016	1,984	BCG+3Penta+3OPV +3PCV+2RV+MCV
Porth et al., 2019 (26)	Worabe, Ethiopia	Cross-sectional	Questionnaire in 2016	232	BCG+3Penta+3OPV +3PCV+2RV+MCV
Tamirat et al., 2019 (27)	Ethiopia	Secondary analysis	DHS 2016	1,909	BCG+3Penta+3OPV +3PCV+2RV+MCV
Mekonnen et al., 2019 (28)	Minjar-Shenkora district, Ethiopia	Cross-sectional	Questionnaire in 2017	566	BCG+3Penta+3OPV +3PCV+2RV+MCV
Tesfaye et al., 2018 (29)	East Gojam Zone in Northwest, Ethiopia	Cross-sectional	Questionnaire in 2016	846	BCG+3Penta+3OPV +3PCV+2RV+MCV
Tefera et al., 2018 (30)	Worabe town Southern, Ethiopia	Cross-sectional	Questionnaire in 2016	540	BCG+3Penta+3OPV +3PCV+2RV+MCV

Author & date	Place of study	Study Design	Data Sources	Sample size	Vaccines assessed in the published literature
Animaw et al., 2014 (31)	Arba Minch town Southern, Ethiopia	Cross-sectional	Questionnaire in 2013	630	BCG+3Penta+3OPV +3PCV+MCV
Moyer et al., 2013 (32)	Ethiopia	Secondary analysis	DHS 2011	3,334	BCG+3Penta+4OPV MCV
Etana et al., 2012 (33)	Ambo Woreda, Central, Ethiopia	Cross-sectional	Questionnaire in 2011	536	BCG+3Penta+3OPV +MCV
Luman et al., 2007 (34)	Ambo and Yaya-Gulelena D/Libanos, Ethiopia	Cross-sectional	Survey in 2003	869	BCG+3Penta+3OPV +3PCV+2RV+MCV
Mmanga et al., 2022 (35)	Malawi	Secondary analysis	DHS 2004, 2010 and 2015-2016	9,177	BCG+3Penta+3OPV +3PCV+2RV+MCV
Johns et al., 2022 (36)	Rural, Malawi	Secondary analysis	DHS 2015-2016	2,740	BCG+3Penta+3OPV +3PCV+2RV+MCV
Ntenda et al., 2019 (37)	Malawi	Secondary analysis	DHS 2015-2016	3,111	BCG+3Penta+3OPV +3PCV+2RV+MCV
Tsega et al., 2016 (38)	Dowa and Ntchisi districts, Malawi	Cross-sectional	Standard WHO EPI cluster survey in 2015	601	BCG+3Penta+3OPV +3PCV+2RV+MCV
Fatiregun et al., 2014 (39)	IBNE and Ido in Southwest, Nigeria	Cross-sectional	Questionnaire in 2012	1,178	BCG+3DTP+3OPV+ 3HepB+MCV+YF
Fatiregun et al., 2012	Umunneochi district of Abia State,	Cross-sectional	Questionnaire in 2010	525	BCG+3DTP+4OPV+ MCV

Author & date	Place of study	Study Design	Data Sources	Sample size	Vaccines assessed in the published literature
(40)	Southern, Nigeria				
Sadoh et al., 2009 (41)	Benin city, Nigeria	Cross-sectional	Questionnaire in 2005	512	BCG+3DTP+3OPV+3HepB+YF+MCV
Allan et al., 2021(42)	Kenya	Secondary analysis	DHS 2014	3,965	BCG+3Penta+3OPV+3PCV+MCV
Maina et al., 2013 (43)	Nakuru and Kaptembwo, Kenya	Cross-sectional	Questionnaire in 2011	380	BCG+3Penta+4OPV+MCV
Kawakatsu et al., 2012 (44)	Siaya, Ugenya, Gem and Kisumu Western, Kenya	Cross-sectional	Questionnaire in 2005	2,560	BCG+3DTP+3OPV+3HepB+MCV
Koulidiati et al., 2022 (45)	Burkina Faso	Cross-sectional	Survey in 2017	3,138	BCG+3Penta+3OPV+3PCV+2RV+MCV
Sia et al., 2009 (46)	Rural, Burkina Faso	Secondary analysis	DHS 1998-1999 and 2003	805	BCG+3Penta+3OPV+YF+MCV
Mukanda et al., 2022 (47)	Mabanga area in Goma city, DRC	Cross-sectional	Questionnaire in 2021	423	BCG+3Penta+3OPV+3PCV+YF+MCV+IPV
Lu et al., 2021 (48)	DRC	Secondary analysis	MICS in 2018	3,524	BCG+3Penta+3OPV+3PCV+2RV+MCV+YF
Shemwell et al., 2017 (49)	Gurue and Milange Districts, Mozambique	Cross-sectional	Survey in 2014	1,650	BCG+3DTP-HepB+4OPV+3PCV+2RV+MCV+3Hib
Jani et al., 2008 (50)	Magude district,	Cross-sectional	Questionnaire in 2001	668	BCG+3Penta+3OPV+MCV

Author & date	Place of study	Study Design	Data Sources	Sample size	Vaccines assessed in the published literature
	southern, Mozambique				
Fisker et al., 2014 (51)	Rural, Guinea-Bissau	Cohort	Follow-up visit in 2007 and 2009	2,701	BCG+3Penta+3OPV +YF+MCV
Hornshoj et al., 2012 (52)	Rural, Guinea-Bissau	Cohort	Follow-up visit in 2005	5,806	BCG+3DTP+3OPV+ MCV
Budu et al., 2020 (53)	Ghana	Secondary analysis	DHS 1998, 2003, 2008, 2014	5,119	BCG+3Penta+3OPV +3PCV+2RV+MCV
Batalingaya a et al., 2023 (54)	Central Africa Republic	Secondary analysis	MICS 2018-2019	1,685	BCG+3Penta+3OPV +3PCV+YF+MCV
Sangaré et al., 2021 (55)	Segou, Mali	Cross-sectional	Questionnaire in 2020	540	BCG+3Penta+3OPV +3PCV+2RV+MCV+ YF+MenA
Montwedi et al., 2021 (56)	Tshwane region 5, gauteng province, South Africa	Cross-sectional	Questionnaire in 2017	216	BCG+3Penta (DTaP-IPV-Hib)+2OPV+3PCV+ 3HepB+2RV+MCV
Budu et al., 2021 (57)	Benin	Secondary analysis	DHS 2018	4,156	BCG+3Penta+3OPV +3PCV+2RV+MCV
Jama et al., 2020 (58)	South Galkayo district, Somalia	Cross-sectional	Questionnaire in 2018	357	BCG+3DPT+4OPV+ 3PCV+2RV+MCV+3 HepB
Cortaredon a et al., 2020 (59)	Senegal	Secondary analysis	DHS 2015-2017	4,955	BCG+3Penta+3OPV +YF+MCV
Ekouevi et al., 2018 (60)	Togo	Cross-sectional	Questionnaire in 2017	1,128	BCG+3Penta+3OPV +3PCV+2RV+MCV

Author & date	Place of study	Study Design	Data Sources	Sample size	Vaccines assessed in the published literature
Russo et al., 2015 (61)	Dschang, West Region, Cameroon	Cross-sectional	Questionnaire in 2013	540	BCG+3Penta+3OPV +MCV+YF
Kruger et al., 2014 (62)	Rural Mbulu area in northern, Tanzanian	Cross-sectional	Questionnaire in 1998, 1999, 2006 and 2007	3,868	BCG+3DTwP-HepB+4OPV+MCV
Babirye et al., 2012 (63)	Kampala, Urban Uganda	Cross-sectional	Questionnaire in 2010	821	BCG+3Penta+4OPV +MCV
Mukungwa et al., 2015 (64)	Zimbabwe	Secondary analysis	DHS 2010-2011	979	BCG+3Penta+3OPV +MCV

Table S2. Overall VSC rate and series completion rate of different vaccines in SSA

Commented [M2]: We renumbered the table. Please confirm.

Author & date	Overall VSC rate	BCG	DPT 3	Polio 3	RV 2	PCV 3	MCV 1
Sako et al., 2023 (12)	39.09%	70.24%	57.85%	55.75%	60.62%	55.16%	57.04%
Gelagay et al., 2023 (13)	30.90%	81.40%	63.60%	64.90%	72.20%	63.60%	60.90%
Worku et al., 2022 (14)	57.76%	90.97%	85.19%	90.15%	86.76%	85.59%	86.41%
Muluye et al., 2022 (15)	50.70%	65.80%	55.60%	60.30%	59.40%	55.50%	61.80%
Darebo et al., 2022 (16)	47.00%	88.70%	88.70%	88.70%	88.70%	88.70%	59.40%
Asmare et al., 2022 (17)	66.1%	88.00%	70.90%	70.90%	77.00%	70.90%	66.10%
Mekonnen et al., 2021 (18)	Invention group vs control group: 82.6% vs 70.9%		95.80% vs 86.90%				91.50 vs 79.30%
Miretu et al., 2021 (19)	57.4%	91.10%	69.50%	69.50%	69.20%	69.50%	78.20%
Jimma et al., 2021 (20)	77.17%	32.00%	76.30%	76.30%	80.40%	76.30%	71.80%
Gelagay et al., 2021 (21)	76.50%	97.10%	91.60%	91.10%	95.00%	89.60%	82.40%
Mekonnen et al., 2020 (22)	64.3%	95.40%	83.20%	80.40%	91.20%	82.20%	76.20%

Author & date	Overall VSC rate	BCG	DPT 3	Polio 3	RV 2	PCV 3	MCV 1
Geweniger et al., 2020 (23)	33.30%	69.20%	53.20%	56.40%	56.00%	49.10%	54.30%
Debie et al., 2020 (24)[a]	24.60% in 2011, 39.00% in 2016						
Debie et al., 2020 (25)[a]	39.00%						
Porth et al., 2019 (26)	75.00%	98.70%	86.58%	94.37%	94.40%	85.78%	84.05%
Tamirat et al., 2019 (27)	38.3%		56.10%	60.40%	58.00%	51.90%	57.80%
Mekonnen et al., 2019 (28)	75.60%	93.50%	92.80%	92.90%	89.20%	91.90%	85.00%
Tesfaye et al., 2018 (29)	58.40%	77.70%	81.30%	82.70%	83.50%	80.60%	81.80%
Tefera et al., 2018 (30)[a]	61.00%						
Animaw et al., 2014 (31)	73.20%	91.00%	89.80%	86.30%		86.30%	77.60%
Moyer et al., 2013 (32)	25.90%	64.30%	35.40%	48.00%			54.50%
Etana et al., 2012 (33)	35.6%	71.10%	47.90%	54,30%			54.90%
Luman et al., 2007 (34)	9.6%	19.20% in Ambo, 51.00% in Yaya,	16.40% in Ambo, 47.40% in Yaya,	11.20% in Ambo, 42.00% in Yaya,			9.20% in Ambo, 39.50% in Yaya,

Author & date	Overall VSC rate	BCG	DPT 3	Polio 3	RV 2	PCV 3	MCV 1
Mmanga et al., 2022 (35)[a]	65% (2004), 84% (2010), 73% (2015-2016)						
Johns et al., 2022 (36)	50.40%	97.50%	93.40%	82.20%	91.10%	89.70%	91.70%
Ntenda et al., 2019 (37)[a]	72.00%						
Tsega et al., 2016 (38)	87% in Dowa, 88% in Ntchisi	99.00% in Dowa, 100.00% in Ntchisi	98.00% in Dowa, 98.00% in Ntchisi	96.00% in Dowa, 93.00% in Ntchisi	96.00% in Dowa, 97.00% in Ntchisi	98.00% in Dowa, 97.00% in Ntchisi	94.00% in Dowa, 93.00% in Ntchisi
Fatiregun et al., 2014 (39)	40.2% in the IBNE and 41.3% in the Ido	76.30%	50.80%	73.70%			42.00%
Fatiregun et al., 2012 (40)	32.40%	59.20%	68.00%	66.00%			76.00%
Sadoh et al., 2009 (41)	44.30%	88.30%	69.50%	59.60%			57.60%
Allan et al., 2021(42)	68.20%	96.70%	90.40%	82.40%		86.00%	87.00%
Maina et al., 2013 (43)	76.6%	99.50%	90.00%	90.50%			77.40%
Kawakatsu et al., 2012 (44)	79.4%	95.90%	87.40%	89.60%			90.20%
Koulidiati et al., 2022 (45)	36.59%	98.00%	92.64%	91.21%	90.57%	90.48%	52.84%

Author & date	Overall VSC rate	BCG	DPT 3	Polio 3	RV 2	PCV 3	MCV 1
Sia et al., 2009 (46)[a]	25.9% in 1998, 41.2% in 2003						
Mukanda et al., 2022 (47)	96.70%	100.00%	99.50%	99.00%	99.70%	99.50%	96.90%
Lu et al., 2021 (48)	36.6%	73.20%	47.30%	69.80%		45.80%	56.90%
Shemwell et al., 2017 (49)	49.7% in Gurue, 48.0% in Milange	97.50% in Gurue, 99.00% in Milange	85.30% in Gurue, 78.20% in Milange	91.40% in Gurue, 83.80% in Milange		63.10% in Gurue, 66.00% in Milange	87.00% in Gurue, 74.70% in Milange
Jani et al., 2008 (50)[a]	71.70%						
Fisker et al., 2014 (51)	53% in 2007, 53% in 2009	89.00% in 2007, 91.00% in 2009	73.00% in 2007, 81.00% in 2009	68.00% in 2007, 78.00% in 2009			71.00% in 2007, 66.00% in 2009
Hornshoj et al., 2012 (52)	50%, 9% (include opv0)	89.00%	68.00%	66.00%			61.00%
Budu et al., 2020 (53)	85.18% in 1998; 85.57% in 2003; 91.60% in 2008; 95.16% in 2014	74.61%	50.63%	50.63%			62.34%
Batalingaya et al., 2023 (54)[a]	65.10%						
Sangaré et al., 2021	81.54%						

Author & date	Overall VSC rate	BCG	DPT 3	Polio 3	RV 2	PCV 3	MCV 1
(55)[a]							
Montwedi et al., 2021 (56)	78.60%	99.30%	95.70%	97.10%	97.10%	87.30%	92.80%
Budu et al., 2021 (57)[a]	85.40%						
Jama et al., 2020 (58)	20.00%	42.00%	28.00%	22.00%			33.00%
Cortaredona et al., 2020 (59)[a]	68.00%						
Ekouevi et al., 2018 (60)	72.32%	90.72%	81.21%	85.25%	86.52%	84.85%	80.89%
Russo et al., 2015 (61)	85.9%	99.80%	94.80%	95.00%			91.20%
Kruger et al., 2014 (62)	71.6% in 1998, 58.0% in 1999, 58.5% in 2006, 57.2% in 2007	98.70% in 1998, 99.10% in 1999, 86.40% in 2006, 90.20% in 2007	84.2% in 1998, 81.0% in 1999, 84.3% in 2006, 78.3% in 2007	85.1% in 1998, 67.2% in 1999, 83.7% in 2006, 84.1% in 2007			72.4% in 1998, 72.7% in 1999, 61.6% in 2006, 58.5% in 2007
Babirye et al., 2012 (63)	77.2%	99.00%	89.10%	89.00%			80.60%
Mukungwa et al., 2015 (64)	65.40%	88.00%	75.00%	75.00%			80.20%

[a] Series completion rate not provided by paper.
